# Psychometric evaluation of the patient-reported experience of cognitive impairment in schizophrenia (PRECIS) scale

**DOI:** 10.1186/s41687-024-00731-x

**Published:** 2024-06-17

**Authors:** William R. Lenderking, Mark J. Atkinson, Mary Kate Ladd, Yulia Savva, Stephanie Sommer, Matthew Sidovar, Claudia Hastedt

**Affiliations:** 1 Patient-Centered Research, Evidera, Bethesda, MD USA; 2 Formerly at Evidera, Bethesda, USA; 3 Boehringer Ingelheim International GmbH, Ingelheim am Rhein, Germany; 4 Formerly at Boehringer Ingelheim Pharmaceuticals, Inc., Ridgefield, USA

**Keywords:** Cognitive impairment associated with schizophrenia (CIAS), Patient-reported experience of cognitive impairment in schizophrenia (PRECIS), Psychometric validation, Factor analysis, Cognitive symptoms

## Abstract

**Background:**

Cognitive impairment associated with schizophrenia (CIAS) represents a distinct, persistent, and core group of schizophrenia symptoms. Cognitive symptoms have been shown to have an impact on quality of life. There are several published CIAS measures, but none based on direct patient self-report. It is important to capture the patient’s perspective to supplement performancebased outcome measures of cognition to provide a complete picture of the patient’s experience. This paper describes additional validation work on the Patient-Reported Experience of Cognitive Impairment in Schizophrenia (PRECIS) instrument.

**Methods:**

Data from two large, international, pharmaceutical clinical trials in medically and psychiatrically stable English-speaking patients with schizophrenia and 88 healthy controls were analyzed. An exploratory factor analysis (EFA) was conducted in one trial (*n* = 215), using the original 35-item PRECIS. The factor structure suggested by EFA was further evaluated using item response theory (IRT; Samejima’s graded response model), and tested using confirmatory factor analysis (CFA). Both EFA and CFA results were tested in a second trial with similar inclusion/exclusion characteristics (*n* = 410). Additional statistical properties were evaluated in healthy controls.

**Results:**

EFA suggested that the best solution after item reduction suggested a factor structure of 6 factors based on 26 items (memory, communication, self-control, executive function, attention, sharpness of thought), supporting a total score, with an additional 2-item bother score (28 items in all). IRT analysis indicated the items were well-ordered within each domain. The CFA demonstrated excellent model fit, accounting for 69% of the variance. The statistical properties of the 28-item version of the PRECIS were confirmed in the second trial. Evidence for internal consistency and test-retest reliability was robust. Known-groups validity was supported by comparison of healthy controls with patients with schizophrenia. Correlations indicated moderate associations between PRECIS and functioning instruments like the Schizophrenia Cognition Rating Scale (SCoRS), but weak correlations with performance-based outcomes like MATRICS Consensus Cognitive Battery (MCCB).

**Discussion:**

Using two clinical trial samples, we identified a robust factor structure for the PRECIS and were able to replicate it in the second sample. Evaluation of the meaningful score difference (MSD) should be repeated in future studies, as these samples did not show enough change for it to be evaluated.

**Conclusions:**

This analysis provides strong evidence for the reliability and validity of the PRECIS, a 28-item, patient-reported instrument to assess cognitive impairment associated with schizophrenia. The correlation with functioning and the weak correlation with performance on cognitive tasks suggests that patient reports of cognitive impairment measure a unique aspect of patient experience.

## Introduction

Cognitive impairments associated with schizophrenia (CIAS) are collectively one of the core symptoms of schizophrenia and can be observed beginning in the prodromal phase of the disease, and may persist even throughout stable periods when patients are not experiencing psychotic symptoms [1]. Cognitive impairments experienced by patients with schizophrenia often include the following: deficits in working and long-term memory, speed of processing, executive function, attention, social cognition, and higherorder problem solving [2]. CIAS has a significant impact on a patient’s quality of life and may interfere with their ability to manage day-to-day tasks. Previous research has shown that CIAS is a stronger predictor of functional impairment than positive or negative symptoms of schizophrenia [3]. However, the relationship between cognitive impairment and functioning is indirect and complex [4]. To fully understand the burden of schizophrenia, it is important to evaluate the patient’s experience with cognitive functioning.

There are currently no approved treatments for CIAS, but clinical trials are ongoing and there are a number of psychometrically validated performance-based and clinician-reported measures that have been used to assess cognition in this patient population (e.g., MATRICS Consensus Cognitive Battery [MCCB] [5, 6]; Cambridge Neuropsychological Test Automated Battery [CANTAB] [7]; Brief Assessment of Cognition [8]) and cognitive functioning (Schizophrenia Cognition Rating Scale [SCoRS] [9]). However, there are no multidimensional patient-reported outcome (PRO) measures that use patients’ self-report to directly assess their experience of CIAS.

This self-assessment gap might be due to the questionable value of self-reports in neuropsychiatric conditions like schizophrenia where disease symptoms may interfere with insight into cognitive difficulties, life function, and overall evaluation of the quality of their lives [10, 11]. There is, nevertheless, a general consensus that it is important and meaningful to incorporate the patient’s direct report of their experience, particularly if patients are medically and mentally stable [12].

While objective clinical assessments may be more reliable across all phases of illness, their validity is sometimes questioned in terms of the meaningfulness of the deficits measured to the patient. Thus, subjective, patient-reported experience is important to examine, in addition to performance-based and clinician-reported assessments, to provide a more complete picture of cognitive functioning. For these reasons, the Food and Drug Administration (FDA) has emphasized the importance of incorporating the patient voice and patient experience into drug development and clinical trials, through the patient-focused drug development initiative [13]. In parallel, the European Medicines Agency is increasingly taking patient experience into consideration for regulatory decision making [14].

In order to better assess patient experience with CIAS, a new measure was developed according to the FDA guidance for instrument development [15]. The development and validation of the initial PatientReported Experience of Cognitive Impairment in Schizophrenia (PRECIS) measure has been previously published [16–18]. The objective of this paper is to briefly summarize the development and content validity of the initial 35-item version of PRECIS, to describe the item-reduction process that led to a refined PRECIS structure, and to explore the psychometric properties of the modified version of PRECIS. The item reduction and psychometric analyses were conducted using data from two Boehringer Ingelheim clinical trials.

## Methods

The PRECIS was developed according to the FDA guidance for instrument development [15]. Briefly, a conceptual model was developed based on a review of the literature and input from clinical advisors. Then qualitative interviews were conducted with patients (*n* = 80) to assess the initial conceptual framework and elicit concepts for draft items based on this framework. The items were worded based on the language used in the concept elicitation interviews. The draft items were reviewed during cognitive debriefing interviews with patients, which resulted in a 35-item draft PRO measure. The initial 35-item multidimensional measure included assessment of seven domains: memory, communication, control, planning, handling problems, attention, and sharp thinking. In addition to the seven domains, there were two final questions that assessed the overall level of bother. All items were answered using a 5-point Likert scale, with higher scores corresponding to worse patient experience (1 = not at all/not at all hard, 2 = a little bit/a little bit hard, 3 = somewhat/somewhat hard, 4 = quite a bit/quite hard, 5 = very much/very hard). All questions used a one-week recall period, and the initial PRECIS 35-item version was scored by summing the 33 items of the seven domains and dividing by 33. The two additional items that were summarizing the overall level of bother with all domains were scored separately, because they are related to the totality of items of all domains. The full details of the development process have been previously published [18].

To evaluate the factor structure of the PRECIS and psychometric properties, data from the Trial 1346.9 “A Phase II Randomized, Double-blinded, Placebo-controlled Parallel Group Trial to Examine the Efficacy and Safety of 4 Oral Doses of BI 425809 Once Daily Over 12 Week Treatment Period in Patients with Schizophrenia” (referred to as Trial 1 throughout) were utilized. Results from Trial 1 describing the safety and efficacy of BI 425,809 have been previously published [19]. The trial included 509 patients; however, the analyses presented here were limited to randomized patients from US sites who had evaluable measurements on any of the PRECIS variables (as the PRECIS was only available in English at the time of the trial, *n* = 215). Participants were adult outpatients with schizophrenia, who were clinically stable, with no hospitalization for worsening of schizophrenia within six months, who were medically stable over four weeks, and psychiatrically stable without symptom exacerbation within three months prior to randomization. The full list of inclusion/exclusion criteria can be found in the clinical trial study publication [19].

First, item descriptive statistics were assessed for all 35-items of the PRECIS (mean, standard deviation, floor/ceiling effects, and percentage of missing response). Then, an exploratory factor analysis [EFA] was run specifying a 6-factor model, based on the analytic procedures of the original validation study [17]. Two confirmatory factor analyses (CFAs) were conducted: the first to confirm the existence of an overall single factor model and support use of a total score for the PRECIS; and a second to confirm a more granular factor structure resulting from the multi-dimensional EFA results. The comparative fit index (CFI; minimum threshold of 0.9) and root mean square error of approximation (RMSEA; maximum acceptable threshold of 0.09) were examined to evaluate the factor structure.

Both the EFA and CFA procedures were repeated using data from Trial 1289.6, “A Phase II Randomised, Double-blind, Placebo-controlled Study to Evaluate the Efficacy, Safety, and Tolerability of Four Orally Administrated Doses of BI 409306 During a 12-week Treatment Period in Patients with Schizophrenia on Stable Antipsychotic Treatment” (referred to as Trial 2 throughout) [20]. Patients were adult patients diagnosed with schizophrenia from 6 countries, and clinically and medically stable for 8 weeks prior to randomization. The details of inclusion/exclusion criteria have been previously published [20].

Once the multi-dimensional factor structure was identified and weaker items were removed (< 0.4 factor loading on any factor), item response theory (IRT) analyses were conducted using data from Baseline (Day 1, prior to first dose). Samejima’s graded response models were fitted to the data separately using items for each factor. Item characteristic curves were developed to verify the correct ordering of the item response options.

In order to design the scoring rules to handle missing data, items within each domain were sequentially removed (strongest to weakest) from internal consistency analyses to determine the number of missing items that could be allowed without reducing the internal consistency or remaining items to an alpha below the commonly acceptable threshold of 0.70.

Finally, the reliability, and validity for the revised PRECIS scales were explored using data from Trial (1) The internal consistency reliability, Cronbach’s alpha and item-total correlations were assessed at Baseline and Week 12. To evaluate test-retest reliability, it is necessary for the retested patients to be stable with regards to the construct being measured by the questionnaire. Therefore, the test-retest reliabilities of PRECIS total and domain scores over time were evaluated using participants in the placebo group who were stable (no change on the Clinician Global Impression of Severity (CGI-S) from Baseline to Week 12. Because of the very long time interval between measurements (12 weeks; see Table 1), test-retest reliability was also evaluated using data with a shorter retest window from Trial (2) In this analysis, stability was defined as no change on the CANTAB (< 1 point change in either direction) from Week 6 to Week 12, and change in PRECIS total score was examined with a 3-week interval between measurements at Week 9 and Week 12.

**Table 1:** Summary of clinical outcome assessment timepoints from Trial 1 and Trial 2

Trial Period	Screening	Randomized Treatment				End of Treatment
**Trial 1**						
**Week**	**-4 to -1**	**1**	**3**	**6**	**9**	**12**
MCCB	✓	✓		✓		✓
SCoRS		✓				✓
CGI-S		✓				✓
PRECIS		✓				✓
PSP		✓				✓
EQ-5D-5 L		✓				✓
**Trial 2**						
**Week**	**-4 to -1**	**1**	**3**	**6**	**9**	**12**
CANTAB	✓	✓		✓		✓
MCCB	✓	✓		✓		✓
SCoRS		✓				✓
CGI-S		✓				✓
PRECIS		✓		✓	✓	✓

CGI-S, Clinician Global Impression of Severity; EQ-5D-5 L, EuroQOL 5 Dimension 5 Level; MCCB, MATRICS Consensus Cognitive Battery; PRECIS, Patient-Reported Experience of Cognitive Impairment in Schizophrenia; PSP, Personal and Social Performance Scale; SCoRS, Schizophrenia Cognition Rating Scale

CANTAB, Cambridge Neuropsychological Test Automated Battery; CGI-S, Clinician Global Impression of Severity; MCCB, MATRICS Consensus Cognitive Battery; PRECIS, Patient-Reported Experience of Cognitive Impairment in Schizophrenia; SCoRS, Schizophrenia Cognition Rating Scale

The construct validity of the PRECIS total score and domain scores were evaluated by their correlations (Spearman’s correlations) with other valid cognitive measures initially using data from Trial 1. It was hypothesized that the PRECIS would have moderate positive correlations with the SCoRS and MCCB, and small, negative correlations with the EQ-5D and PSP.

Known-groups validity was assessed by stratifying participants into groups according to the MCCB overall and neurocognition scores (1 SD below normative mean < [40] vs. normal or above [≥ 40]), as well as the CGI-S score groups (normal, borderline, or mildly ill vs. markedly, severely, or most extremely ill). Known-groups validity was examined at Baseline and Week 12, using a fixed-group analysis of variance (ANOVA) with post-hoc category comparisons via Tukey’s test to determine if the PRECIS scores statistically differed between groups.

Known-groups validity was also assessed using data from Trial 2 and a separate, parallel study with 88 healthy controls. The healthy controls were recruited from the same clinical sites used in Trial 2, and were recruited to match demographic characteristics (e.g., gender, age) of the participants, and were without major psychiatric illness, neuropsychological impairment or a history of antipsychotic drug use. PRECIS total and domain median scores were analyzed using a Mann-Whitney U test to compare the clinical and normal control groups.

## Results

### Trial 1 baseline descriptive statistics

At Baseline, the full range of response options was represented for all PRECIS items (range = 1–5). Higher scores correspond to worse patient experience, and the mean scores for the 35 items ranged from 1.6 to 2.6 (Table 2). On all but two items, 30% or more of the sample responded with a 1 (not at all/not at all hard), indicating a potential ceiling effect. This is perhaps not surprising, since not all patients experience all of the cognitive impairments described by PRECIS items. Since only 5.1% of the participants reported a 1 for all PRECIS items (data on file), most study participants endorsed at least some cognitive difficulties.

**Table 2:** 35-Item PRECIS: Descriptive statistics at baseline day 1 (*n* = 215)

PRECIS-35*	Mean (SD); Range	% Ceiling (1)	% Floor (5)	% Missing
Item 1 – Recall Peoples Names^†^	2.0 (1.1); 1.0–5.0	97 (45.1%)	3 (1.4%)	0 (0.0%)
Item 2 – Remember Things to Do or Buy	1.9 (0.9); 1.0–5.0	88 (40.9%)	1 (0.5%)	0 (0.0%)
Item 3 – Remember Where Things Were Put	2.0 (1.0); 1.0–5.0	86 (40.0%)	3 (1.4%)	0 (0.0%)
Item 4 – Remember Recent Information	2.1 (1.1); 1.0–5.0	79 (36.7%)	7 (3.3%)	0 (0.0%)
Item 5 – Recall Something from Years Ago^†^	2.2 (1.2); 1.0–5.0	75 (34.9%)	12 (5.6%)	0 (0.0%)
Item 6 – Remember What to Say	1.9 (1.0); 1.0–5.0	90 (41.9%)	3 (1.4%)	0 (0.0%)
Item 7 – Remember What Someone Else Said	2.0 (1.0); 1.0–5.0	74 (34.4%)	7 (3.3%)	0 (0.0%)
Item 8 – Remember How to Get Somewhere	1.7 (1.0); 1.0–5.0	127 (59.1%)	6 (2.8%)	0 (0.0%)
Item 9 – Remember What I Was About to Do	1.8 (1.0); 1.0–5.0	110 (51.2%)	1 (0.5%)	0 (0.0%)
Item 10 – Understand What Someone Was Saying^†^	1.8 (1.0); 1.0–5.0	107 (49.8%)	6 (2.8%)	0 (0.0%)
Item 11 – Say Something When I Wanted	1.9 (1.0); 1.0–5.0	95 (44.2%)	8 (3.7%)	0 (0.0%)
Item 12 – Interact with People	2.3 (1.3); 1.0–5.0	79 (36.7%)	23 (10.7%)	0 (0.0%)
Item 13 – Explaining What I Meant	2.0 (1.1); 1.0–5.0	93 (43.3%)	9 (4.2%)	0 (0.0%)
Item 14 – Finding Words to Say What I Meant	2.0 (1.1); 1.0–5.0	92 (42.8%)	7 (3.3%)	0 (0.0%)
Item 15 – Understand Non-Verbal Gestures^†^	1.8 (1.1); 1.0–5.0	113 (52.6%)	8 (3.7%)	0 (0.0%)
Item 16 – Keep Things from Slipping Out	1.8 (1.1); 1.0–5.0	110 (51.2%)	6 (2.8%)	0 (0.0%)
Item 17 – Think Through Before Speaking/Doing	1.9 (1.1); 1.0–5.0	109 (50.7%)	11 (5.1%)	0 (0.0%)
Item 18 – Stop Saying/Doing Something Wrong	1.6 (1.0); 1.0–5.0	128 (59.5%)	5 (2.3%)	0 (0.0%)
Item 19 – Plan Ahead Without Someone’s Help	1.8 (1.1); 1.0–5.0	112 (52.1%)	8 (3.7%)	0 (0.0%)
Item 20 – Things Do Not Happen as Usual^†^	2.1 (1.1); 1.0–5.0	77 (35.8%)	10 (4.7%)	0 (0.0%)
Item 21 – Someone Changed Plans Last Minute	2.2 (1.3); 1.0–5.0	86 (40.0%)	17 (7.9%)	0 (0.0%)
Item 22 – Come Up with Solutions To Problems	1.9 (1.0); 1.0–5.0	101 (47.0%)	7 (3.3%)	0 (0.0%)
Item 23 – Coming Up with New or Different Way	1.9 (1.0); 1.0–5.0	97 (45.1%)	5 (2.3%)	0 (0.0%)
Item 24 – Understand Effect of Now and Future^†^	2.0 (1.1); 1.0–5.0	93 (43.3%)	8 (3.7%)	0 (0.0%)
Item 25 – Mind Drifted Paying Attention	2.6 (1.3); 1.0–5.0	48 (22.3%)	24 (11.2%)	0 (0.0%)
Item 26 - Distracted by My Surroundings	2.5 (1.2); 1.0–5.0	51 (23.7%)	11 (5.1%)	0 (0.0%)
Item 27 – Hard to Stay on Task	2.3 (1.2); 1.0–5.0	78 (36.3%)	9 (4.2%)	0 (0.0%)
Item 28 – Kept Thinking About Things	2.4 (1.3); 1.0–5.0	75 (34.9%)	19 (8.8%)	0 (0.0%)
Item 29 – Thoughts Were Racing and Speeding	2.1 (1.2); 1.0–5.0	93 (43.3%)	13 (6.0%)	0 (0.0%)
Item 30 – Thinking was Unclear/Cloudy/Foggy	2.2 (1.1); 1.0–5.0	72 (33.5%)	12 (5.6%)	0 (0.0%)
Item 31 – Not Thinking as Fast as Others	2.4 (1.3); 1.0–5.0	70 (32.6%)	19 (8.8%)	0 (0.0%)
Item 32 – Thoughts Were Slower Than I Wanted	2.3 (1.2); 1.0–5.0	75 (34.9%)	14 (6.5%)	0 (0.0%)
Item 33 – It Was Hard to Think What to Say	2.0 (1.3); 1.0–5.0	108 (50.2%)	18 (8.4%)	0 (0.0%)
Item 34 – Bothersome Experiences with Thinking	1.9 (1.2); 1.0–5.0	106 (49.3%)	10 (4.7%)	0 (0.0%)
Item 35 – Bothersome if Thinking Stayed the Same	2.4 (1.4); 1.0–5.0	77 (35.8%)	25 (11.6%)	0 (0.0%)

PRECIS, Patient-Reported Experience of Cognitive Impairment in Schizophrenia; SD, standard deviation

*Items were re-numbered for 28-item version

^†^Item deleted based on the results of the factor analysis

Ceiling = PRECIS Score of 1 (not at all/not at all hard); Floor = PRECIS Score of 5 (very much/very hard)

### Factor analysis and item reduction

An exploratory factor analysis was run specifying a 6-factor model, based on the findings from the original validation study [17]. In the 6-factor model, two domains overlapped on one factor. As additional multi-factor solutions were explored, there was a gain in the proportion of variance explained in the 7-factor solution. The 7-factor solution was used to remove weakly loading items (< 0.4 factor loading on any factor) (Table 3). The factor containing two general items related to the overall level of bother was dropped from the calculation of the 7-factor solution, thereby providing a final 6-factor model (memory, communication, self-control, executive function, attention, sharpness of thought) contributing to the total. The two bother items, although still part of the measure, are analyzed separately because they were designed to assess the general degree to which the various individual concepts measured by the scale overall mattered to the patient, and are not a measure of cognitive functioning per se. The final 6-factor model had good model fit (CFI = 0.925; RMSEA = 0.045).

**Table 3:** Exploratory factor analysis: 7-factor principal component factors

PRECIS Item	Number PRECIS-35	Factor Loadings						
		F1	F2	F3	F4	F5	F6	F7
**Memory**								
Recall Peoples Names^†^	CIAS101	0.41	0.29	0.03	-0.03	0.32	-0.09	-0.16
Remember Things to Do or Buy	CIAS102	0.01	**0.65**	0.24	-0.22	0.09	0.03	0.10
Remember Where Things Were Put	CIAS103	0.06	**0.57**	0.14	-0.07	0.14	-0.03	-0.08
Remember Recent Information^†^	CIAS104	0.40	**0.44**	0.20	-0.04	0.03	-0.15	-0.04
Recall Something from Years Ago^†^	CIAS105	0.44	0.20	0.23	0.01	0.17	-0.12	-0.30
Remember What to Say	CIAS106	0.14	**0.53**	0.14	0.09	-0.05	0.03	-0.01
Remember What Someone Else Said	CIAS107	0.02	**0.57**	0.07	0.27	0.11	-0.08	-0.05
Remember How to Get Somewhere	CIAS108	0.07	**0.78**	-0.22	-0.18	-0.07	0.24	0.16
Remember What I Was About to Do	CIAS109	-0.04	**0.89**	-0.18	0.03	-0.01	0.00	0.12
Understand What Someone Was Saying^†^	CIAS110	-0.12	0.42	-0.07	0.51	-0.03	-0.08	0.23
**Communication**								
Say Something When I Wanted	CIAS111	**0.74**	0.06	-0.03	0.25	-0.27	0.02	0.07
Interact with People	CIAS112	**0.80**	-0.15	-0.04	-0.02	0.11	0.18	-0.08
Explaining What I Meant	CIAS113	**0.65**	0.19	0.12	-0.04	-0.29	0.16	0.10
Finding Words to Say What I Meant	CIAS114	**0.68**	0.20	0.01	0.07	-0.26	0.13	0.06
Understand Non-Verbal Gestures^†^	CIAS115	**0.45**	0.19	-0.26	0.03	0.23	0.09	0.05
**Self-control**								
Keep Things from Slipping Out	CIAS116	0.09	-0.05	0.02	**0.76**	-0.06	0.09	-0.06
Think Through Before Speaking/Doing	CIAS117	0.15	0.04	-0.21	**0.63**	0.22	-0.02	0.12
Stop Saying/Doing Something Wrong	CIAS118	0.12	-0.16	0.03	**0.85**	-0.09	0.04	0.07
**Executive Function**								
Plan Ahead Without Someone’s Help	CIAS119	0.06	0.15	0.20	-0.04	-0.12	**0.58**	0.15
Things Do Not Happen as Usual^†^	CIAS120	0.37	-0.07	-0.19	0.01	0.42	0.28	0.14
Someone Changed Plans Last Minute	CIAS121	0.13	-0.11	-0.15	-0.09	0.41	**0.57**	0.15
Come Up with Solutions to Problems	CIAS122	0.16	0.04	0.18	0.08	-0.09	**0.72**	-0.11
Coming Up with New or Different Way	CIAS123	0.11	-0.03	0.25	0.08	-0.06	**0.74**	-0.13
Understand Effect of Now on Future^†^	CIAS124	-0.28	0.17	0.14	0.39	0.22	**0.49**	-0.25
**Attention**								
Mind Drifted Paying Attention	CIAS125	-0.02	-0.04	**0.78**	-0.05	0.16	0.06	0.08
Distracted by My Surroundings	CIAS126	0.04	-0.01	**0.66**	-0.11	0.05	0.23	0.10
Hard Staying on Track	CIAS127	-0.11	0.06	**0.75**	0.02	0.07	0.24	-0.05
Kept Thinking About Things	CIAS128	0.33	-0.40	**0.50**	0.06	0.25	-0.09	0.18
Thoughts Were Racing and Speeding	CIAS129	0.24	-0.17	**0.47**	0.08	0.07	-0.06	0.17
Thinking was Unclear/Cloudy/Foggy	CIAS130	-0.06	0.02	**0.64**	0.03	-0.10	0.06	0.35
**Sharpness of Thought**								
Not Thinking as Fast as Others	CIAS131	-0.02	0.10	0.26	0.02	0.15	-0.06	**0.61**
Thoughts Were Slower Than I Wanted	CIAS132	0.00	0.02	0.13	-0.01	0.13	0.10	**0.66**
It Was Hard to Think What to Say	CIAS133	0.01	0.19	0.08	0.10	-0.03	-0.14	**0.76**
**Bother Items**								
Bothersome Experiences with Thinking	CIAS134	-0.25	0.23	0.14	0.13	**0.60**	-0.05	0.15
Bothersome if Thinking Stayed Same	CIAS135	-0.21	0.01	0.23	-0.05	**0.88**	-0.03	0.03

PRECIS, Patient-Reported Experience of Cognitive Impairment in Schizophrenia

^†^Items were removed due to poor loadings

Bolded entries are the strongest loadings on the hypothesized factor

Following item reduction, all of the PRECIS items’ factor loadings on their factors were well above the minimum loading threshold of 0.40, ranging from 0.64 to 0.87 on their own factors, with moderate but lower correlations with all other factors. The final 26-item, 6-factor solution explained 69% of the variance. Based on a confirmatory factor analysis, the new factor structure had excellent model fit (comparative fit index [CFI] > 0.9; root mean square error of approximation *≤* 0.05 with a confidence interval [17] between 0.0 and 1.0; and a standardized root mean squared residual *≤* 0.08) (Table 4). The final structure of the PRECIS was then determined to be 26 items on 6-factors, with two additional items assessing the overall level of bother that were not included in any of the domain scores nor the total score. This is referred to as the 28-item PRECIS.

**Table 4:** Confirmatory factor analysis: Factor loadings for hierarchical (Second-order) 6-factor model and 1-factor model (Trial 1346.9)

PRECIS	Factor Loadings for PRECIS Domains for 6-Factor Model						Factor Loadings for 1Factor Model
	Memory	Communication	Self-control	Executive Function	Attention	Sharpness of Thought	
**Memory**							
Remember Things to Do or Buy	0.755	-	-	-	-	-	0.692
Remember Where Things Were Put	0.636	-	-	-	-	-	0.582
Remember What to Say	0.752	-	-	-	-	-	0.677
Remember What Someone Else Said	0.756	-	-	-	-	-	0.682
Remember How to Get Somewhere	0.752	-	-	-	-	-	0.606
Remember What I Was About to Do	0.665	-	-	-	-	-	0.613
**Communication**							
Say Something When I Wanted	-	0.810	-	-	-	-	0.687
Interact with People	-	0.687	-	-	-	-	0.638
Explaining What I Meant	-	0.799	-	-	-	-	0.746
Finding Words to Say What I Meant	-	0.787	-	-	-	-	0.731
**Self-control**							
Keep Things from Slipping Out	-	-	0.755	-	-	-	0.564
Think Through Before Speaking/Doing	-	-	0.850	-	-	-	0.649
Stop Saying/Doing Something Wrong	-	-	0.831	-	-	-	0.614
**Executive Function**							
Plan Ahead Without Someone’s Help	-	-	-	0.853	-	-	0.723
Someone Changed Plans Last Minute	-	-	-	0.637	-	-	0.593
Come Up with Solutions to Problems	-	-	-	0.840	-	-	0.727
Coming Up with New or Different Way	-	-	-	0.764	-	-	0.705
**Attention**							
Mind Drifted Paying Attention	-	-	-	-	0.783	-	0.726
Distracted by My Surroundings	-	-	-	-	0.786	-	0.718
Hard Staying on Track	-	-	-	-	0.805	-	0.735
Kept Thinking About Things	-	-	-	-	0.661	-	0.611
Thoughts Were Racing and Speeding	-	-	-	-	0.650	-	0.601
Thinking was Unclear/Cloudy/Foggy	-	-	-	-	0.761	-	0.705
**Sharpness of Thought**							
Not Thinking as Fast as Others	-	-	-	-	-	0.870	0.733
Thoughts Were Slower Than I Wanted	-	-	-	-	-	0.820	0.691
It Was Hard to Think What to Say	-	-	-	-	-	0.768	0.656
**2nd Order factor loadings on total cognitive symptoms**	**0.871**	**0.922**	**0.805**	**0.899**	**0.869**	**0.811**	

CFI, comparative fit index; CI, confidence interval; df, degrees of freedom; PRECIS, Patient-Reported Experience of Cognitive Impairment in Schizophrenia; RMSEA, root mean square error of approximation; SD, standard deviation; SRMR, standardized root mean squared residual

Two bother items not included in scoring

For 6-Factor Model: Chi-square (df) = 422.58 (293); CFI = 0.925; RMSEA = 0.045, 90% CI for RMSEA = 0.035–0.055; SRMR = 0.042

A CFA was conducted using data from Trial 2, which replicated the factor structure of the 28-item PRECIS (these CFA results are presented in the Appendix A).

### Item performance

IRT analysis utilized data from Baseline to evaluate the performance of the items in each of the 6 PRECIS factors. Samejima’s graded response model was used to evaluate the performance of each item. Item characteristic curves, slopes and threshold parameters were evaluated. Overall, there was no evidence to suggest that the items’ response options were out of order. However, there was some indication, based on the z-score statistics, that the lowest response category for a number of items was not significantly different than the next higher response category (e.g., ‘no’ vs. ‘little’ cognitive symptomatology over the last week). For example, this was the case for 3-items in the communication domain (Say Something When I Wanted; Explaining What I Meant; Finding Words to Say What I Meant; *p* > 0.05). For no item in the PRECIS was there more than one significant z-score difference across the response options, indicating good separation between the response options.

### PRECIS scoring

The CFA for the one-factor model confirmed that a total score can be calculated, with acceptable factor loadings (> 0.55) for all items. Based on a recursive procedure to decide how many items can be missing and still result in the remaining items possessing an alpha > 0.70, it was determined that 19 of the 26 items must be available to calculate the total scoring. When calculating domain scores, each of the domains can have the following number of missing items and still be scored: Memory:3; Communication:1; Self-control:0; Executive function:0; Attention:3; Sharpness of thought:0.

### Reliability

Cronbach’s alpha and item-total correlations with that item deleted were assessed for PRECIS scores at Baseline and Week 12 in Trial 1. Cronbach’s alpha for the 28-item PRECIS was excellent (α = 0.95 and 0.96 for Baseline and Week 12, respectively). Cronbach’s alpha of the domain scores were also high, ranging from 0.88 (attention) to 0.79 (self-control) at Baseline.

To assess test-retest reliability, PRECIS scores were compared at Baseline and Week 12 for subjects in the placebo group that had a stable CGI-S score across the timepoints in Trial 1. For the 38 subjects in this population, the ICC approached the target of 0.70, with an ICC of 0.64 for the total PRECIS score. This may be due to the long timeframe between measurements that was not ideal for test-retest reliability. This analysis was repeated using data from Trial 2, where PRECIS was assessed in shorter time intervals, and where placebo patients were defined as stable if they had no change on the CANTAB from Week 6 to Week 12. For these stable subjects, ICCs were calculated for a 3-week interval (between the PRECIS total score at Week 9 and Week 12). The ICC was 0.77, exceeding the target of 0.70.

### Validity

At Baseline in Trial 1, the PRECIS total score and domain scores showed significant low to moderate correlations with the SCoRS (0.64 for the total score; domains ranged from 0.45 to 0.57), the EQ-5D (Index: -0.44 for the total score; domains ranged from − 0.33 to −0.39; visual analog scale (VAS): −0.33 for the total score, domains ranged from − 0.24 to −0.33), and the Personal and Social Performance Scale (PSP) [21] (0.19 for the total score). Similar low to moderate correlations were seen at Week 12 (Table 5). Correlations with the MCCB overall and neurocognitive scores were poor and not significant.

**Table 5:** Construct validity: Spearman correlations between PRECIS scores and other measures at baseline (*n*=215)

Measure	PRECIS Correlation Coefficient							
	Total Score (26 items)	Memory	Communication	Self-Control	Executive Function	Attention	Sharpness of Thought	Bother Items (mean)
**Baseline Day 1**								
MCCB overall composite	0.06	0.05	0.07	-0.03	0.02	0.11	0.04	0.09
MCCB neurocognitive domain	0.07	0.06	0.08	-0.02	0.05	0.11	0.05	0.08
SCoRS total score	0.64****	0.57****	0.57****	0.45****	0.50****	0.54****	0.54****	0.40****
SCoRS global rating score	0.42****	0.39****	0.34****	0.30****	0.31****	0.37****	0.40****	0.29****
EQ-5D-5 L Index score	-0.44****	-0.37****	-0.38****	-0.38****	-0.38****	-0.39****	-0.33****	-0.39****
EQ-5D-5 L VAS	-0.33****	-0.33****	-0.25***	-0.29****	-0.29****	-0.24***	-0.32****	-0.36****
PSP	-0.19**	-0.21**	-0.16*	-0.14*	-0.10	-0.16*	-0.11	-0.19**
**EoT Week 12**								
MCCB overall composite	0.12	0.13	0.10	0.03	0.03	0.19*	0.02	0.11
MCCB neurocognitive domain	0.14	0.13	0.11	0.05	0.06	0.21*	0.05	0.12
SCoRS total score	0.62****	0.58****	0.52****	0.39****	0.55****	0.58****	0.48****	0.53****
SCoRS global rating score	0.42****	0.39****	0.34****	0.23*	0.43****	0.38****	0.34****	0.36****
EQ-5D-5 L Index score	-0.39****	-0.36****	-0.38****	-0.39****	-0.34****	-0.36****	-0.28***	-0.37****
EQ-5D-5 L VAS	-0.25***	-0.24***	-0.25***	-0.22**	-0.20**	-0.23**	-0.16*	-0.25***
PSP	-0.25***	-0.21**	-0.24***	-0.19**	-0.28****	-0.24**	-0.17*	-0.30****

EoT, end of treatment; EQ-5D-5 L, EuroQol-5 Dimensions-5 Levels (higher score = better health); MCCB, MATRICS Consensus Cognitive Battery (higher score = better cognitive abilities); PRECIS, Patient-Reported Experience of Cognitive Impairment in Schizophrenia (higher score = more impairment); PSP, Personal and Social Performance (higher score = worse performance); SCoRS, Schizophrenia Cognition Rating Scale (higher score = more impairment); VAS, visual analog scale

Spearman rank-order correlations; * *p* < 0.05; ** *p* < 0.01; *** *p* < 0.001; **** *p* < 0.0001

For known-groups validity, participants were stratified by MCCB overall and neurocognitive scores, and CGI-S scores in Trial 1. The PRECIS total score distinguished between subjects grouped by CGI-S score at Baseline (F = 5.35, *p* < 0.05) and Week 12 (F = 9.35, *p* < 0.05). However, the PRECIS total score was not able to distinguish between groups (below norm [< 40] vs. normal or above [≥ 40]) based on the MCCB overall score at Baseline (2.1(0.78) vs. 2.0(0.71)) or Week 12 (1.9(0.76) vs. 1.9(0.70)) (*p* > 0.05). Similarly, the PRECIS neurocognition score did not distinguish between groups.

When comparing between subjects in Trial 2 and healthy controls, the PRECIS domain and total scores were all significantly different between groups (*p* < 0.001; Table 6).

**Table 6:** Known-groups validity: PRECIS scores for trial 1289.6 patients and healthy controls at baseline

Domain	Healthy Controls *N* = 88 Median (Q1– Q3)	Trial 1289.6 Subjects *N* = 410 Median (Q1– Q3)	*p*-value^a^
Memory	1.25 (1.00–1.50)	1.67 (1.17–2.33)	< 0.001
Communication	1.00 (1.00–1.50)	1.75 (1.25–2.50)	< 0.001
Self-control	1.00 (1.00–1.30)	1.50 (1.00–2.00)	< 0.001
Executive Function	1.25 (1.00–1.50)	1.75 (1.25–2.50)	< 0.001
Attention	1.50 (1.33–1.83)	2.17 (1.50–3.17)	< 0.001
Sharpness of Thought	1.33 (1.00–1.33)	2.00 (1.33–2.67)	< 0.001
Bother Items	1.00 (1.00–2.00)	2.00 (1.50–3.00)	< 0.001
PRECIS Total Score	1.29 (1.16–1.55)	1.96 (1.50–2.50)	< 0.001

^a^*p*-value based on non-parametric test (Mann-Whitney U Test)

## Discussion

The results presented in this paper add to the evidence to date suggesting that the PRECIS is a reliable and valid PRO well-suited to assessing change in patient-reported experience with cognitive impairment associated with schizophrenia. This finding is important, as existing performance-based measures do not directly capture the patient-reported burden experienced with cognitive functioning. Furthermore, there is a dearth of patient-reported instruments suitable for patients with schizophrenia to assess their own cognitive functioning and how they experience it in their day-to-day lives. The PRECIS was developed following a rigorous process involving qualitative research with patients and psychometric validation using data from two sizable clinical trials. It has been re-conceptualized as an assessment of 6 factors of cognition, and the item reduction has lessened the burden to patients.

The revised structure of the 28-item PRECIS described in this paper has excellent model fit, and the factor analysis demonstrated that responses can be scored by domain as well as summed to an overall total score. This structure also demonstrated very good internal consistency reliability for the individual domains and the total score.

The PRECIS total score and domain scores had low to moderate correlations with the SCoRS and the PSP. The SCoRS is an interview-based and rater-assessed measure of cognitive functioning, and the PSP is a clinician rating of functioning. The findings were expected, given the overlap between subjective experience with cognitive functioning as measured by the PRECIS and functioning as measured by the rater assessed instruments SCoRS and PSP. While all of these instruments assess related aspects of functioning, the PRECIS is distinct in that the patient is assessing their own internal feelings, thoughts, and awareness of their thoughts, which external raters, who focus on observed behavior cannot fully assess. The PRECIS did not significantly correlate with the MCCB test battery, which is an objective, performance-based measure of cognition. This demonstrates that how a patient feels about his or her cognitive abilities and how well they function in daily life do not appear to directly relate to how they perform on cognitive tasks. Furthermore, the tasks and specific abilities measured with standardized tasks in the MCCB capture cognitive performance, but this may not be directly related to a patient’s ability to complete day to day tasks and function in their real life. This supports conclusions from a semi-systematic review which detailed an indirect association between cognition and functioning, with many mediating and moderating factors [4]. This suggests that there is value in measuring the patient’s perspective on their own cognitive functioning among people living with schizophrenia, as what is being measured by the PRECIS is a different domain than objectively measured cognition.

The correlation between the EQ-5D-5 L and the PRECIS was moderate, suggesting that the subjective experience with cognitive impairment assessed by PRECIS plays a role in patients’ overall health related quality of life, but PRECIS also assesses (disease) specific aspects not captured by generic quality of life instruments. Overall, the results of the construct validity analysis suggest that the PRECIS measures subjective perceptions of cognitive functioning, which are not directly related to objective cognitive measures, although related to clinician ratings of cognitive functioning, and also related to quality of life. Therefore, it is an important complement to objective cognitive impairment measures and standard quality of life questionnaires.

One limitation regarding the patient population should be acknowledged. In order to be eligible to participate in the trial, participants were required to be medically and psychiatrically stable. As a result, the population included in this analysis did not report high levels of cognitive impairment at Baseline, as measured by the PRECIS. At Baseline, 50% of the participants had a PRECIS total score less than two (2 = a little bit/a little bit hard), which indicated little perceived overall cognitive impairment. Additionally, 26 of the 28 retained PRECIS items demonstrated 30% or more of the sample reporting ‘no impairment’ on these individual items, indicating a ceiling effect (no further room for improvement). However, only 5.1% of participants responded with a one (1 = not at all/not at all hard) for all items of the PRECIS.

The full range of response options was represented for all PRECIS items, and the total score ranged from 1.0 to 4.6, indicating that a broad range of levels of cognitive impairment were included. Given the breadth and variability of cognitive functioning, we would not expect participants to report experiencing difficulty with all of the concepts described in the PRECIS items, so this finding is not altogether unexpected. Nevertheless, in the future, it will be important to evaluate the PRECIS in a population of patients with greater variation in their levels of cognitive impairment. In addition to evaluating the PRECIS in a more varied patient population, future work is also needed to evaluate the threshold for meaningful within-patient change on the PRECIS, and the ability of the PRECIS to detect change. The measure is currently being translated into several additional languages following guidelines for linguistic validation [22].

## Conclusions

This analysis provides strong evidence for the reliability and validity of the PRECIS, a 28-item, patient-reported instrument to assess cognitive impairment associated with schizophrenia.

The correlation with functioning and the weak correlation with performance on cognitive tasks suggests that patient reports of cognitive impairment measure a unique aspect of patient experience. The results presented here, in addition to the rigorous development work for the PRECIS that has been previously published, demonstrate that the PRECIS is a valid PRO with robust psychometric properties, and is well-suited to assessing patient-reported change in perceived cognitive impairment.

## Data Availability

The datasets analyzed during the current study are available from the corresponding author on reasonable request.

## Appendix A

**Table A1:** Confirmatory factor analysis: factor loadings for hierarchical (second order) 6-factor model (trial 1289.6)

PRECIS	Factor Loadings for PRECIS Domains					
	Memory	Communication	Self-control	Executive Function	Attention	Sharpness of Thought
**Memory**						
Remember Things to Do or Buy	0.735	-	-	-	-	-
Remember Where Things Were Put	0.670	-	-	-	-	-
Remember What to Say	0.715	-	-	-	-	-
Remember What Someone Else Said	0.774	-	-	-	-	-
Remember How to Get Somewhere	0.752	-	-	-	-	-
Remember What I Was About to Do	0.721	-	-	-	-	-
**Communication**						
Say Something When I Wanted	-	0.764	-	-	-	-
Interact with People	-	0.711	-	-	-	-
Explaining What I Meant	-	0.712	-	-	-	-
Finding Words to Say What I Meant	-	0.775	-	-	-	-
**Self-control**						
Keep Things from Slipping Out	-	-	0.751	-	-	-
Think Through Before Speaking/Doing	-	-	0.855	-	-	-
Stop Saying/Doing Something Wrong	-	-	0.772	-	-	-
**Executive Function**						
Plan Ahead Without Someone’s Help	-	-	-	0.743	-	-
Someone Changed Plans Last Minute	-	-	-	0.600	-	-
Come Up with Solutions to Problems	-	-	-	0.865	-	-
Coming Up with New or Different Way	-	-	-	0.846	-	-
**Attention**						
Mind Drifted Paying Attention	-	-	-	-	0.763	-
Distracted by My Surroundings	-	-	-	-	0.726	-
Hard Staying on Track	-	-	-	-	0.834	-
Kept Thinking About Things	-	-	-	-	0.745	-
Thoughts Were Racing and Speeding	-	-	-	-	0.710	-
Thinking was Unclear/Cloudy/Foggy	-	-	-	-	0.778	-
**Sharpness of Thought**						
Not Thinking as Fast as Others	-	-	-	-	-	0.773
Thoughts Were Slower Than I Wanted	-	-	-	-	-	0.726
It Was Hard to Think What to Say	-	-	-	-	-	0.810
**2nd Order factor loadings on total cognitive symptoms**	0.863	0.889	0.786	0.876	0.858	0.81

CFI, comparative fit index; CI, confidence interval; df, degrees of freedom; PRECIS, Patient-Reported Experience of Cognitive Impairment in Schizophrenia; RMSEA, root mean square error of approximation; SRMR, standardized root mean squared residual

Chi-square (df) = 589.8.0 (293); CFI = 0.918; RMSEA = 0.052, 90% CI for RMSEA = 0.046–0.058; SRMR = 0.044

## Abbreviations

ANCOVA: Analysis of covariance
CANTAB: Cambridge Neuropsychological Test Automated Battery
CFA: Confirmatory factor analyses
CFI: Comparative fit index
CGI-S: Clinician Global Impression of Severity
CIAS: Cognitive impairments associated with schizophrenia
EFA: Exploratory factor analysis
FDA: Food and Drug Administration
ICC: Intra-class correlation coefficient
IRT: Item response theory
MCCB: MATRICS Consensus Cognitive Battery
PRECIS: Patient-Reported Experience of Cognitive Impairment in Schizophrenia
PRO: Patient-reported outcome
PSP: Personal and Social Performance Scale
SCoRS: Schizophrenia Cognition Rating Scale
VAS: Visual analog scale
